# Osteogenesis and neurogenesis: a robust link also for language evolution

**DOI:** 10.3389/fncel.2015.00291

**Published:** 2015-07-28

**Authors:** Cedric Boeckx, Antonio Benítez-Burraco

**Affiliations:** ^1^Catalan Institute for Advanced Studies and ResearchBarcelona, Spain; ^2^Linguistics, Universitat de BarcelonaBarcelona, Spain; ^3^Spanish Philology and its Didactics, University of HuelvaHuelva, Spain

**Keywords:** osteogenesis, neurogenesis, modern cognition, language evolution, RUNX2

This paper seeks to contribute to the characterization of the relation between osteogenesis and neurogenesis by approaching it from the field of the neurobiology of language and cognition; specifically, from an evolutionary perspective. It is difficult to ascertain how the hominin brain changed to support modern language and cognitive abilities because we can only rely on skull remains. But insights can be gained from fossils because the brain and the skull exhibit a tight relationship. Skull shape and brain shape and connectivity influence one another (Roberts et al., 2010; Lieberman, 2011). Craniofacial anomalies and cognitive disorders frequently co-occur (see Boeckx and Benítez-Burraco, 2014a for review). So, “osteo” considerations can shed light on “neuro” considerations (and vice versa). Importantly, main differences between anatomically-modern humans (AMHs) and Neanderthals pertain not to the brain size, but to the more globularized headshape of the former (Bruner, 2004). Globularity results from an AMH-specific developmental trajectory after birth, at a stage when the brain is the primary determinant of skull shape (Gunz et al., 2010). Globularization is not just a morphological change of the skull. On the contrary, factors giving rise to globularity also have important neurofunctional consequences. The hypothesis we have explored in our recent work is that the rewiring of the hominin brain associated to globularization brought about our most distinctive mode of cognition (see Boeckx and Benítez-Burraco, 2014a for details).

In a series of related papers (Boeckx and Benítez-Burraco, 2014a,b; Benítez-Burraco and Boeckx, 2015) we have examined closely some of the most critical genes that may contribute to skull globularity and that have been selected in AMHs. These also contribute significantly to neurogenesis, as well as to neural specification, arealization of the neo-cortex, neuronal interconnection, and synaptic plasticity. Eventually, the very osteogenic signals that help build our distinctive skull also contributes to build our distinctive mode of brain organization underlying our mode of cognition and language abilities.

Our main candidate is *RUNX2*. A selective sweep in this gene occurred after our split from Neanderthals (Green et al., 2010). It is a candidate for cleidocranial dysplasia (Yoshida et al., 2003) and controls the closure of cranial sutures (Stein et al., 2004). Together with *DLX5* and *TLE1* it regulates the integration of the parietal bone (Depew et al., 1999; Stephens, 2006), a “hotspot” for globularization (Bruner, 2004). However, it is also involved in the development of the hippocampal GABAergic neurons as part of the GAD67 regulatory network (Pleasure et al., 2000; Benes et al., 2007). Moreover, it seems to be also involved in the development of thalamus (Reale et al., 2013). Its mutations cause mental diseases in which our mode of cognition is impaired (Talkowski et al., 2012; Ruzicka et al., 2015). Importantly, *RUNX2* is deeply implicated in the regulation of osteocalcin (Paredes et al., 2004) and osteopontin (Shen and Christakos, 2005), which are important for both bone formation and brain organization (e.g., osteopontin-deficient mice suffer from thalamic neurodegeneration; Schroeter et al., 2006).

Interestingly, *RUNX2* is functionally connected to many genes that are important for brain and language development, but also to bone formation. To begin with, *RUNX2* is a regulatory target of AUTS2 (Oksenberg et al., 2014). *AUTS2* is among the genes found to be differentially expressed after *RUNX2* transfection in neuroblastomic cell lines (Kuhlwilm et al., 2013). The first half of *AUTS2* displays the strongest signal of positive selection in AMHs compared to Neanderthals (Green et al., 2010). Mutations in *AUTS2* give rise to a host of cognitive impairments (see Oksenberg and Ahituv, 2013 for review). Interestingly, these routinely co-occur with skeletal abnormalities and/or dysmorphic features (Beunders et al., 2013). AUTS2 interacts with some other proteins like TBR1, RELN, SATB2, GTF2I, ZMAT3, or PRC1 that play a key role at the brain level and have been related to ASD and other developmental disorders affecting cognition and language (Oksenberg and Ahituv, 2013). Some of them directly interact with RUNX2.

For example, *RUNX2* directly interacts with *SATB2* (Hassan et al., 2010), a gene that regulates stereotypic projections in the cortex (Srinivasan et al., 2012). This gene has been related to ASD, intellectual disability, and language delays, as well as craniofacial defects (Liedén et al., 2014) and plays a key role in osteoblast differentiation, palate formation, and craniofacial development (Zhao et al., 2014). Crucially, the interaction between SATB2 and *RUNX2* is very relevant during osteogenesis (Hassan et al., 2010; Gong et al., 2014). Specifically, several micro-RNAs (including miR-205 and miR-31), SATB2, RUNX2, osteopontin and osteocalcin interact complexly to modulate the differentiation of bone mesenchymal stem cells into osteoblasts (Deng et al., 2013; Hu et al., 2015). Interestingly, in the neural *satb2* expression depends on both Bmp and Shh (Sheehan-Rooney et al., 2013), which are genes we have highlighted in our previous work. Moreover, SATB2 represses the expression of *HOXA2* (Ye et al., 2011), which is one of the targets of the famous “language gene” *FOXP2* (Konopka et al., 2009). *HOX2A* is involved in both the brain and bone formation. Accordingly, it contributes to the hindbrain patterning (Miguez et al., 2012), acting upstream the guidance signals Robo1, Robo2, Slit1, and Slit2 in the anteroposterior migration of pontine neurons (Geisen et al., 2008). However, it also encodes an inhibitor of bone formation (Dobreva et al., 2006; Ye et al., 2011), which controls the morphology of the skeleton (Tavella and Bobola, 2010). Interestingly also, the activation of *Hoxa2* in the neural crest downregulates Bmp antagonists and leads to severe craniofacial and brain defects (Garcez et al., 2014).

Additionally, RUNX2 interacts (via FOXO1) with *DYRK1A* (Huang and Tindall, 2007), a gene located within the Down Syndrome Critical Region on chromosome 21. This gene has been linked to microcephaly, facial dysmorphism, mental retardation, and absence of speech (van Bon et al., 2011; Courcet et al., 2012). *DYRK1A* has been shown to be involved in bone homeostasis as an inhibitor of osteoclastogenesis (Lee et al., 2009). DYRK1A is also of interest because it phosphorylates SIRT1, which controls neural precursor activity and differentiation (Saharan et al., 2013). SIRT1 both upregulates *RUNX2* and deacetylates RUNX2, ultimately promoting osteoblast differentiation (Shakibaei et al., 2012; Srivastava et al., 2012), an effect which is also due to its effects on β-catenin and FoxO in osteoblast progenitors (Iyer et al., 2014). Importantly, resveratrol-induced SIRT1 activation promotes neuronal differentiation of human bone marrow mesenchymal stem cells (Joe et al., 2015). Finally, *RUNX2* is also functionally related (via AUTS2) to *CBL*, in turn linked to Noonan syndrome-like disorder, a condition involving facial dysmorphism, a reduced growth, and several cognitive deficits (Martinelli et al., 2010). This gene, which encodes an inhibitor of osteoblast differentiation and promotes the degradation of Osterix (Choi et al., 2015), is located within a region showing signals of a strong selective sweep in AMHs compared to Altai Neanderthals (Prüfer et al., 2014).

RUNX2 is also functionally directly linked to the FOXP2 and ROBO1 interactomes (see Boeckx and Benítez-Burraco, 2014b for details), which are related to language disorders and vocal learning (Graham and Fisher, 2013; Pfenning et al., 2014). To begin with, a direct interaction between *RUNX2* and *FOXP2* has recently been experimentally demonstrated (Zhao et al., 2015b). This finding was further reinforced in Gascoyne et al. (2015), who added *FOXP2* to the list of established osteoblast and chondrocyte transcription factors such *RUNX2, SP7*, and *SOX9*. In fact, *FOXP2* seems to regulate both bone formation (it regulates endochondral ossification) (Zhao et al., 2015b), and the fate of neural stem cells during corticogenesis (MuhChyi et al., 2013). As for the ROBO suite, some members like *HES1* and *AKT1* are functionally related to RUNX2. *HES1* is needed for the correct functioning of the Slit/Robo signaling pathway during neurogenesis (Borrell et al., 2012) and plays a role as well in the development of both GABAergic and dopaminergic neurons. *Hes1* silencing promotes bone marrow mesenchymal stem cells to differentiate into GABAergic neuron-like cells *in vitro* (Long et al., 2013). Moreover, Hes1 modulates skeletal formation and pathogenesis of osteoarthritis via calcium/calmodulin interaction (Sugita et al., 2015). In turn *AKT1* is a critical mediator of growth factor-induced neuronal survival (Dudek et al., 1997). In mice mutations in *Akt1* and *Akt2* impair bone formation (Peng et al., 2003). *AKT1* has recently been shown to coordinate the bone-forming osteoblasts and bone-resorbing osteoclasts, a process important for maintaining skeletal integrity (Akt1 deficiency impairs osteoclast differentiation and diminishes the rate of proliferation of osteoblast progenitors) (Mukherjee et al., 2014).

Other bone morphogenetic factors may well play a key role in the emergence of our language-readiness and our globular brain. Among them we wish highlight the DLX suite (particularly, *DLX1, DLX2, DLX5*, and *DLX6*) and the BMP suite (specifically, *BMP2* and *BMP7*): most of them also interact with *RUNX2*. Consider, e.g., *DLX2*. It is involved in craniofacial development (Jeong et al., 2008), but it is also needed for neocortical and thalamic growth (Jones and Rubenstein, 2004). Mutations in this gene affect craniofacial and bone development (Kraus and Lufkin, 2006), but also cognitive development (Liu et al., 2009). It also takes part in the regulation of neuronal proliferation within the cortex (McKinsey et al., 2013). Concerning the BMP proteins, both BMP2 and BMP7 interact with RUNX2 and both of them play a role in bone and brain formation. *BMP2* promotes the differentiation of mesenchymal cells into bone cells (Dwivedi et al., 2012), but it is also needed for normal neurogenesis in the ganglionic eminences and correct cortical neurogenesis (Shakèd et al., 2008). In mice Bmp2 (and also Bmp7) upregulates *Dlx1, Dlx2, Dlx5*, and *Runx2* (Bustos-Valenzuela et al., 2011). Much like *BMP2, BMP7* is involved in osteogenesis (Cheng et al., 2003) and skull and brain development (Segklia et al., 2012). Mutations in this gene give rise as well to developmental delay and learning disabilities (Wyatt et al., 2010).

We further believe that the genetic aspects highlighted here may contribute not only to gain a better understanding of the way in which both aspects of our modernity emerged and interact, but specifically to tune the crosstalk between the osteogenic and neurogenic stem cell niches. Zhao et al. (2015a) have recently identified *Gli1*+ cells within the suture mesenchyme as the main mesenchymal stem cell population for craniofacial bones. Ablation of these *Gli1*+ cells leads to craniosynostosis, known to be associated with cognitive deficits (Starr et al., 2007), and arrest of skull growth. Not surprisingly, Gli1 is known to regulate *Runx2* (Kim et al., 2013). In turn, Gli1 transcriptional activity is regulated by Dyrk1a (Mao et al., 2002), whereas Hes1 directly modulates *Gli1* expression (Schreck et al., 2010). Moreover, *Gli1* is the direct response gene of Shh (Liu et al., 1998). The Shh-Gli1 pathway has been shown to regulate brain growth (Dahmane et al., 2001; Ruiz i Altaba et al., 2002; Corrales et al., 2004), and to control thalamic progenitor identity and nuclei specification (Vue et al., 2009), as well as the development of the cerebellum (Lee et al., 2010). It may also be the case that *FoxP2* lies downstream of *Shh*, as suggested by Scharff and Haesler (2005), who observed that the zinc finger motif of FoxP2 is highly homologous to those of the major Shh downstream transcriptional effectors, particularly, of Gli1, Gli2, and Gli3. Moreover, balanced Shh signaling is required for proper formation and maintenance of dorsal telencephalic midline structure (Himmelstein et al., 2010). Dysregulation of the neural stem cell pathway Shh-Gli1 has been observed in autoimmune encephalomyelitis and multiple sclerosis (Wang et al., 2008). As a matter of fact, a GLI1-p53 inhibitory loop controls neural stem cell (Stecca and Ruiz i Altaba, 2009). Most interestingly for us, Marcucio et al. (2005) have shown that excessive Shh activity, caused by truncating the primary cilia on cranial neural crest cells, causes hypertelorism, and frontonasal dysplasia. This condition has been shown to be associated to mental retardation, lack of language acquisition, and severe central nervous system deficiencies (Guion-Almeida and Richieri-Costa, 2009). The latter example appears to lend credence to our final claim that language and cognition are intimately related to the molecular mechanisms associated with mesenchymal stem cell and neural stem cell populations.

## Conflict of interest statement

The authors declare that the research was conducted in the absence of any commercial or financial relationships that could be construed as a potential conflict of interest.
